# Post-Synthetic Regulation of HS Structure: The Yin and Yang of the Sulfs in Cancer

**DOI:** 10.3389/fonc.2013.00331

**Published:** 2014-01-14

**Authors:** Romain R. Vivès, Amal Seffouh, Hugues Lortat-Jacob

**Affiliations:** ^1^Université Grenoble-Alpes, Institut de Biologie Structurale, Grenoble, France; ^2^CNRS, Institut de Biologie Structurale, Grenoble, France; ^3^CEA, DSV, Institut de Biologie Structurale, Grenoble, France

**Keywords:** glycosaminoglycan, heparan sulfate, interaction, signaling, sulfatase, cancer

## Abstract

Heparan sulfate (HS) is a complex polysaccharide that takes part in most major cellular processes, through its ability to bind and modulate a very large array of proteins. These interactions involve saccharide domains of specific sulfation pattern (S-domains), the assembly of which is tightly orchestrated by a highly regulated biosynthesis machinery. Another level of structural control does also take place at the cell surface, where degrading enzymes further modify HS post-synthetically. Amongst them are the Sulfs, a family of extracellular sulfatases (two isoforms in human) that catalyze the specific 6-O-desulfation of HS. By targeting HS functional sulfated domains, Sulfs dramatically alter its ligand binding properties, thereby modulating a broad range of signaling pathways. Consequently, Sulfs play major roles during development, as well as in tissue homeostasis and repair. Sulfs have also been associated with many pathologies including cancer, but despite increasing interest, the role of Sulfs in tumor development still remains unclear. Studies have been hindered by a poor understanding of the Sulf enzymatic activities and conflicting data have shown either anti-oncogenic or tumor-promoting effects of these enzymes, depending on the tumor models analyzed. These opposite effects clearly illustrate the fine tuning of HS functions by the Sulfs, and the need to clarify the mechanisms involved. In this review, we will detail the present knowledge on the structural and functional properties of the Sulfs, with a special focus on their implication during tumor progression. Finally, we will discuss attempts and perspectives of using the Sulfs as a biomarker of cancer prognosis and diagnostic and as a target for anti-cancer therapies.

## Introduction

In metazoan organisms, coordination of individual cells behavior is largely determined by the concerted action of two large ensembles of pericellular molecules: the extracellular matrix (ECM), which provides cells a solid substratum, and small soluble effector proteins that diffuse within the extracellular milieu and carry signaling activities (1–3). Once released, these diffusible factors may bind to specific signaling receptors expressed at the surface of target cells where they trigger definite biological responses. Most of these extracellular messengers, which include hundreds of interleukins, monokines, lymphokines, chemokines, growth factors, morphogens, …, etc. (and will be collectively referred here as cytokines), are endowed with pleiotropic and overlapping activities. They can be secreted by many distinct cell types, their cognate receptors are widely distributed within tissues, and they are usually effective at very low concentrations (nano- to picomolar range). However, their activities are not systemic and, except during pathological conditions, their functions are strictly focused and highly regulated in time and space (4). For that purpose, regulation of signaling events carried out by diffusible ligands also takes place upstream of the ligand-receptor interaction itself, through processes in which the ECM plays a central role. It has been indeed widely appreciated that the ECM, through which soluble factors diffuse, does not just ensure tissue cohesiveness but by immobilizing many of the above mentioned soluble cytokines provides a structural basis to control their activity. Mechanistically, this sequestration changes their availability, stability, structure, and reactivity, and could lead to specific processing. This provides cells with a system to control and regulate, in their close surrounding, the information carried out by soluble factors (5, 6). It is therefore perhaps not surprising that these soluble and insoluble systems are strongly connected and work together to regulate cellular communication, as the former makes possible the transfer of information between distant cells, while the latter provides a scaffold for multicellularity (7). Amongst ECM components, proteoglycans (PGs) represent one of the major classes of molecules that immobilize and control cytokines (2, 3, 8, 9). PGs bind a variety of growth factors and matrix molecules which play crucial roles in cancer cell-stroma communications (10). The binding of soluble factors to PGs generates reservoirs and gradients and, in promoting ligands receptor recognition, amplifies signaling. In that context, the purpose of this review is to discuss how Sulfs, a recently discovered enzyme family modifying PG structure, take part to the complex relationships between cells and their close surroundings, with a special focus on tumor development.

## Proteoglycans and Glycosaminoglycans

Proteoglycans constitute a group of some 30 heterogeneous glycoproteins that are substituted with specific polysaccharides of the glycosaminoglycan (GAG) family (11). GAGs are anionic and linear polysaccharides, which are ubiquitously present within the ECM and at the surface of virtually all cells. They comprise hyaluronan (which does not occur as a PG but in free form) chondroitin sulfate (CS), dermatan sulfate (DS), heparan sulfate (HS), and heparin. Whereas the core protein usually determines the localization of the molecule, the attached GAG chains, and in particular those of the HS type, are predominantly involved in protein recognition (12, 13). HS binds hundreds of protein ligands, including cytokines (as defined above) but also adhesion and matrix molecules, receptors, enzymes, plasma proteins, etc. These interactions serve a large number of purposes and functionally, HS has been known to affect the local concentration, the compartmentalization, the stability, the structure, and/or the activity of its binding partners. Protein-HS interactions thus play critical roles in a very large number of biological systems, for example, in mediating the formation of chemokine gradients along which cells can migrate directionally (14, 15), in providing a template to juxtapose two proteins such as growth factor-receptor complexes to facilitate signal transduction (16), in protecting cytokines against proteolysis, in inducing protein conformational changes (17), in providing reservoirs of active factors that can be mobilized in specific conditions, or in generating local concentrations of a given binding protein by controlling its diffusion (18) thereby generating local concentrations of a given protein. In particular, as mentioned above, many of the proteins that direct cellular proliferation, such as FGFs (19) and TGF (20), cellular migration, and metastasis such as CXCL12 (21), or control angiogenesis, including VEGF, endostatin, or TG2 (22), are all regulated upon binding to HS in the pericellular milieu.

From a structural view, the multiple binding activities of HS are closely related to its extended structural variability. The chain is synthesized in the Golgi apparatus by enzymes that initially polymerize alternating glucuronic acid (GlcA) and *N*-acetyl-glucosamine (GlcNAc). The resulting disaccharide repeats are then variously modified by interdependent reactions that do not occur uniformly along the chain. The *N*-deacetylase/sulfotransferases NDSTs first catalyze the N-deacetylation, usually followed by the N-sulfation of the GlcNAc units. Remarkably, this occurs in restricted domains of usually 3–6 disaccharides (known as S-domains) in which the GlcA can be C5-epimerized into iduronic acid (IdoA), followed by various O-sulfations, frequently at the C6 and C2 position of the GlcN and IdoA respectively, and more rarely at the C3 and C2 positions of the GlcN and GlcA respectively. Variations in the degree of epimerization and sulfation patterning generate a very large polydispersity and, as such, provide HS chains with distinct docking sites for the various ligands of the polysaccharide (23). Protein binding properties are therefore largely dependent on the degree and pattern of HS sulfation, which are dynamically regulated at the level of tissue and cell type, as well as during development and pathological conditions such as tumor progression. The 6-O-sulfation of HS, for instance, has been shown to be important for binding/activation of many signaling molecules involved in cell proliferation, adhesion, and migration. These include FGFs such as FGF1, FGF4, FGF7 and FGF10 (24–28), HGF (26, 29), VEGF (30), PDGF (31), fibronectin (32), and chemokines such as CXCL12 (33). Furthermore, in the case of FGF2, although 6-*O*-sulfates are not necessary for binding, they are required for promoting the growth factor activity (34–36).

Expectedly, a number of reports have associated increased levels of 6-O-sulfation with tumor progression, highlighting HS biosynthesis 6-*O*-sulfotransferases (6OSTs) as attractive targets for anti-cancer and anti-angiogenic therapies (37, 38). In mammals, three 6OST isoforms have been identified (39). However, these enzymes showed very little differences in substrate specificity, which did not suggest a tight control of HS 6-O-sulfation status during its biosynthesis (40).

## Sulfs: Extracellular Sulfatases Regulating HS Structure and Function

The assembly of specific saccharide motifs involved in protein binding/recognition was originally thought to rely exclusively on the complex and highly regulated HS biosynthesis machinery. The field took a dramatic turn, when Dhoot and colleagues identified in quail a new extracellular sulfatase, QSulf-1, that positively regulated Wnt signaling through desulfation of HSPGs (41). Orthologs have since been identified in human, mouse, rat, chicken, zebra fish, *Drosophila, Xenopus*, and *C. elegans*, as well as two isoforms of the enzyme, Sulf-1 and -2 (42–45). Sulfs were shown to be endosulfatases that catalyzed selective removal of 6-*O*-sulfate groups from internal S-domains in cell surface and ECM HS and rapidly emerged as critical regulators of key functions of the polysaccharide in physiological processes such as embryogenesis and tissue regeneration, and in diseases such as cancer.

Sulfs markedly distinguish from other members of the sulfatase family. Sulfatases are structurally and mechanistically highly conserved enzymes in eukaryotic and prokaryotic species (46). They are intracellular enzymes involved in the catabolism of sulfated compounds and exert their exosulfatase activity in acidic conditions of lysosomal compartments. Their catalytic domain comprises a unique post-translational modification, an α-formylglycine (FGly) residue resulting from oxidation of a strictly conserved cysteine, which is essential for enzyme activity (47). Many members of the sulfatase family are able to cleave sulfate ester from small aryl compounds such as 4-methylumbelliferyl sulfate (4-MUS), and are thus referred to as arylsulfatases (46). In contrast, Sulfs are extracellular enzymes that exert an endosulfatase activity at neutral pH and display poor sequence homology with other sulfatases. Sulf-1 and -2 are very similar in length (870–875 amino acids, depending on species) and are characterized by a unique structural organization. Based on sequence homology, Sulfs consist of four primary domains: a cleavable signal sequence, a catalytic domain (CAT), a central hydrophilic domain (HD), and a C-terminal region (C-ter) (44, 48, 49).

The Sulf CAT domain is highly homologous to the conserved catalytic domains of all eukaryotic sulfatases. It contains the post-translationally modified FGly residue that directly participates in the hydrolytic cleavage reaction of sulfate esters at neutral pH (46, 50). In contrast, the HD domain is a unique feature of the Sulfs and shows no homology with any other protein sequences. It is particularly rich in basic amino acids, including a C-terminal cluster of 12 consecutive basic amino acids, and represents by itself 40% of Sulf overall charge (27% basic and 13% acid). The HD domain is not required for the enzyme arylsulfatase activity and hydrolysis of small substrates, such as 4-MUS. However, it is essential for recognition and endosulfatase activity on HS, and for cell surface localization of the Sulfs (48, 49). Finally, the C-ter domain displays significant homology with the C-ter region of lysosomal glucosamine-6-sulfatase (G6S). It has been suggested that C-ter regions of Sulfs and G6S confer specificity toward glucosamine (44). The mature Sulf consists of a disulfide-linked heterodimer of 75 and 50 kDa subunits, which is formed through processing of a 125-kDa pro-protein by furin-type proteases (49, 51). There are two putative furin-type proteinase cleavage sites in Human Sulf isoforms HSulf-1 and -2. Blocking the cleavage by mutating both sites had no effect on secretion, aryl sulfatase activity, and solubility of Sulfs but inhibited their ability to potentiate Wnt signaling (49). Interestingly, other studies showed that deletion or mutation of furin cleavage sites had no effect on the enzyme endosulfatase activity, or ability to inhibit FGF signaling (48, 51). Finally, Sulfs are glycosylated proteins, with 10–11 putative N-glycosylation sites, mainly located within the N-terminal half of the enzyme (44). Glycosylation inhibitor studies revealed that glycosylation of QSulf-1 is essential for its enzymatic activity, membrane targeting, and secretion (52). However, the role of glycosylation in human Sulfs has not been investigated yet.

The substrate specificity of the Sulfs remains unclear. However, *in vivo* and *in vitro* data indicate that these enzymes essentially exert their 6-*O*-sulfatase activity on (UA2S-GlcNS6S) trisulfated disaccharides units, which are found within HS S-domains, and to a much lesser extent on (UA-GlcNS6S) disulfated disaccharides (53–57). No effect on other 6-*O*-sulfated disaccharides could be observed, suggesting a requirement for *N*-sulfate groups. In contrast, the nature of the uronate (either glucuronic or IdoA) does not seem to be critical for Sulf activity. For QSulfs, 6-O-desulfation was reported on GlcA- but not IdoA-containing disulfated disaccharides (53), but HSulf-2 showed similar activity on both these disaccharides (58). QSulf-1 and -2 have shown similar substrate specificity and redundant HS remodeling functions (53, 57). For mammalian Sulfs, further information about enzyme specificity has been obtained from studying knock-out mice (54, 59–65). Sulf-1 or -2 single knock-out mice showed normal viability and no phenotypic and histological defects, suggesting overlapping functions of the two isoforms and compensation effects. In contrast, Sulf-1/2 double deficient mice suffered multiple developmental abnormalities and high neonatal mortality. Structural analyses of HS from mSulf-1^−/−^ fibroblasts showed elevated levels of all 6-*O*-sulfated disaccharides compared with the wild-type HS. For mSulf-2^−/−^ HS, 6-O-sulfation increase was mainly observed within HS non-sulfated and transition zones (54), indicating that the enzyme may also act outside HS S-domains. These data therefore indicate that, unlike their avian orthologs, mammalian Sulf activity may not be restricted to HS highly sulfated S-domains, and that Sulf-1 and -2 isoforms may have different effects on HS sulfation pattern. In agreement with this, another study on Sulf knock-out mice recently suggested that Sulf-1 and -2 isoforms differentially contributed to the generation of organ-specific sulfation patterns of HS (66).

**Figure 1:**
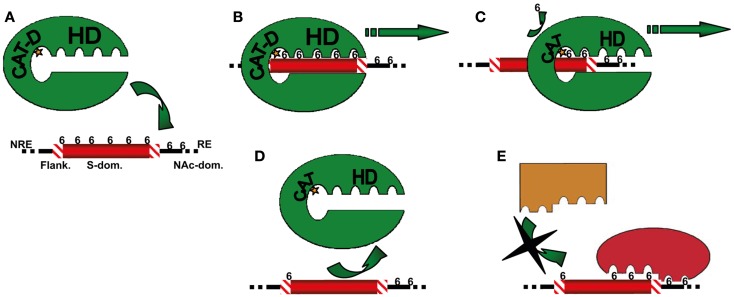
**Model for HSulf processive activity and differential regulation of HS ligand binding properties**. **(A)** HSulf hydrophilic domain (HD) preferentially recognizes and binds to 6-*O*-sulfates harbored by trisulfated disaccharides found within HS S-domains (S-dom.), the catalytic site (CAT-D) of the enzyme being positioned on the most upstream 6-*O*-sulfate residue **(B)**. After desulfation, the enzyme progresses along the polysaccharide chain to accommodate other 6-*O*-sulfate groups **(C)**. Once the S-domain reducing end is reached, the absence of appropriate 6-*O*-sulfates (those present on the flanking regions (Flank.), or the NAc domains (NAc-dom.) are poor substrates for the enzyme) downstream CAT-D results in a strong decrease in the affinity of the enzyme HD for the polysaccharide and the dissociation of the complex **(D)**. Partial and orientated 6-O-desulfation of S-domains may differentially alter HS ligand binding **(E)**. From Seffouh et al. (55), copyright by the Federation of American Societies for Experimental Biology.

The mechanisms by which these enzymes catalyze HS desulfation is poorly understood. The CAT domain, comprising the FGly residue, is able to hydrolyze the general arysulfatase substrate 4-MUS (Km of ≈10 mM). Because of the close homology of this domain with arylsulfatase A, B (ARS-A; ARS-B), and G6S, it is thought that Sulfs share a similar desulfation mechanism. In ARS-A, hydroxylation of the FGly residue by a water molecule forming the activated hydroxyl formylglycine (a formylglycine hydrate) is a necessary step for the enzyme’s sulfatase activity (46). One of the two oxygens of the aldehyde hydrate attacks the sulfur of the sulfate ester, leading to a transesterification of the sulfate group onto the aldehyde hydrate. Simultaneously, the substrate alcohol is released. The released sulfate is eliminated from the enzyme-sulfate intermediate by an intramolecular rearrangement. The “intramolecular hydrolysis” allows the aldehyde group to be regenerated. The active site of ARS-A comprises nine conserved residues that are found to be critical for catalytic activity (67). Based on this information, site-directed mutagenesis should help in understanding whether Sulf-1 and -2 share the same enzymatic mechanism as lysosomal sulfatases. In contrast, the 6-O-desulfation of HS natural substrate requires the presence of the HD domain (48, 49). In a recent study, we have investigated the mechanism by which HSulfs affect S-domain sulfation pattern, using a highly sulfated heparin octasaccharide as a mimic of HS functional S-domains (55). Analyses including mass spectrometry, NMR, and HS oligosaccharide sequencing revealed that HSulfs catalyze the 6-O-desulfation of HS by a non-random, processive, and oriented mechanism, starting from the non-reducing end of the oligosaccharide and progressing throughout the whole polysaccharide toward it reducing end. In addition, we demonstrated that alteration of 6-O-sulfation pattern directed by such mechanism enabled differential regulation of FGF-1 and -2 ligands. From these observations, we proposed a model, in which HSulf CAT domain exerts a non-specific arylsufatase activity, while the HD domain is responsible for substrate specificity and binding, and directs HS processive desulfation (Figure 1). The HD domains of HSulf-1 and -2 display poor sequence identity (44). Differences in activity and ligand binding properties of the two isoforms could therefore result from specific features of their HD domains. This model suggests that HSulf HD domain plays a major role in the enzyme activity and specificity and highlights the need of investigating further its interaction with HS. In this regard, Milz et al. very recently showed that the HD domain indeed conferred HSulf specificity toward 6-*O*-sulfates and that it comprises at least two distinct HS-binding sites that allow for dynamic interaction with the polysaccharide (68).

## Multifaceted Activities of the Sulfs

### A mechanistic view: Sulfs as modulators of growth factor signaling

**Figure 2:**
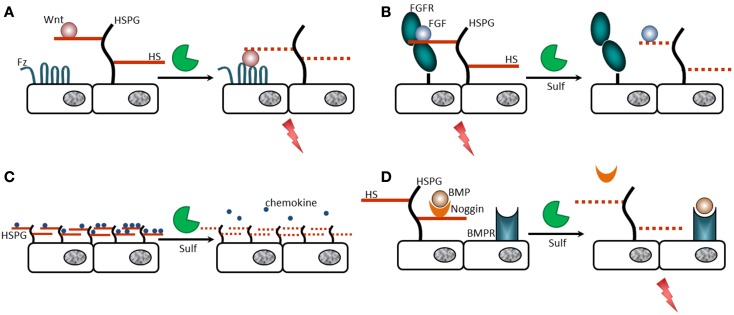
**Regulation of HS ligand binding/activating properties by the Sulfs**. **(A)** High affinity binding of Wnt to 6-*O*-sulfated HS (HS chains shown as plain red lines) prevents interaction to its cell surface receptor Frizzled (Fz). Removal of 6-*O*-sulfates by the Sulfs (HS chains shown as dashed red lines) lowers HS/Wnt affinity, enabling binding of Wnt to Fz and subsequent signaling (red thunderbolt). **(B)** Formation of the functional FGF/FGF receptor (FGFR)/HS ternary complex requires 6-*O*-sulfates. Sulf catalyzed 6-O-desulfation of HS does affect FGF2 binding to HS, but prevents formation of the signaling complex. **(C)** Chemokines/HS interaction is critical for the formation of chemotactic gradients. By inhibiting the interaction of HS with a number of these chemokines, Sulfs may destabilize such gradients. **(D)** Noggin binds with high affinity to 6-*O*-sulfated HS and sequesters BMP. Upon Sulf action, the release of Noggin from the cell surface prevents efficient inhibition of BMP, which can bind to its cognate receptor BMPR and induce signaling.

As discussed above, 6-*O*-sulfates are critical for many biological properties of HS. Through their ability to edit 6-O-sulfation status at the level of HS functional S-domains, Sulfs thus constitute a unique regulatory switch to control ligand binding/activation and subsequent cellular signaling events. As expected, treatment of heparin with Sulfs dramatically reduces its ability to bind a number of protein ligands, including VEGF, FGF1, and chemokines SDF/CXCL12 and SLC in an ELISA assay (69). However and most interestingly, Sulf catalyzed alterations of HS structure cannot simply be associated with loss of function (Figure 2; Table 1). Rather, these will induce some signaling pathways and inhibit others, depending on the isoform involved (Sulf-1 or -2), the protein ligands targeted, and the biological context. As such, QSulf-1 was originally identified for its ability to promote Wnt signaling (41, 53). Indeed, although HS is required for Wnt activity, high affinity interaction with the polysaccharide prevents its binding to the Frizzled (Fz) receptor and subsequent signaling. QSulf-1 induced remodeling of HS 6*-*O-sulfation pattern reduced its affinity for Wnt, enabling formation of a HS/Wnt/Fz functional complex (53). A similar mechanism was reported for the activation of GDNF by Sulf-1 and -2 during mouse neuronal development (59) and maintenance of spermatogonial stem cells (63). Sulfs also enhance BMP signaling by modulating its inhibitor Noggin (57, 70), but on the contrary downregulate FGF1, FGF2, HGF, HB-EGF, VEGF, amphiregulin, and TGFβ signaling (55, 71–76). Finally, Sulf-1 has been shown to either enhance or repress Sonic Hedgehog (Shh) signaling during neuronal development or in gastric cancer, respectively (77, 78).

**Table 1:** **Modulation of HS ligand binding/activating properties by the Sulfs**.

Ligand	Function	Effects of Sulfs		Reference
		Binding	Activity	
Wnt	Embryonic development, cancer	↓	↑	Ai et al. (53), Dhoot et al. (41)
GDNF	Neuronal cell protection/regeneration, spermatogenesis	↓	↑	Ai et al. (59), Langsdorf et al. (63)
BMP	Bone/cartilage morphogenesis		↑	Otsuki et al. (70), Viviano et al. (57)
FGF1	Angiogenesis, wound healing, Embryonic development, cancer	↓	↓	Seffouh et al. (55), Uchimura et al. (69)
FGF2	Angiogenesis, wound healing, Embryonic development, cancer		↓	Dai et al. (71), Frese et al. (48), Lai et al. (72), Li et al. (74), Narita et al. (75)
HGF	Angiogenesis, tissue regeneration, cancer		↓	Lai et al. (73), Narita et al. (75)
HB-EGF	Angiogenesis, wound healing, cancer		↓	Dai et al. (71), Lai et al. (72)
SDF/CXCL12	Chemotaxis, cancer	↓		Uchimura et al. (69)
VEGF	Angiogenesis, cancer	↓	↓	Narita et al. (75), Uchimura et al. (69)
Amphiregulin	Tissue morphogenesis, cancer		↓	Narita et al. (79)
TGFβ	Tissue regeneration, embryonic development, regulation of the immune system, cancer		↓	Yue et al. (76)
Shh	Embryonic development, axonal guidance, cancer		↑ ↓	Danesin et al. (77), Ma et al. (78)

### A functional view: Role of the Sulfs during development

Insights into the physiological functions of the Sulfs have been obtained from the study of knock-out organisms, which highlighted broad and most probably overlapping functions during development. As discussed above, only mSulf-1/2 double knock-out mice display marked phenotypic flaws, which include a variety of renal, lung, skeletal, and neuronal defects (59–61, 64). Amongst these, it is worth noting that bone skeletal defects observed in the Sulf-1^−/−^/Sulf-2^−/−^ embryos show similarities with those reported previously in Hs2st-deficient mice, but also EphrinB1 and BMP-deficient mice and FGFR-1 or -3 hypermorphic mice (60). This phenotype convergence supports further a role of the Sulfs in the regulation of BMP, FGF, and/or Ephrin signaling during sternal development. Neuronal development defects in mSulf knockout mice included neuroanatomical abnormalities and impaired neurite outgrowth, suggesting non-redundant functions of these two enzymes during the development of the nervous system (61). During embryogenesis, Sulfs also promote esophageal innervation, by enhancing GDNF-mediated neurite sprouting (59), and Sulf-1 is involved in the series of events inducing the switch of ventral neural progenitor cells toward an oligodendroglial fate, by modulating Shh distribution and signaling on apical neuroepithelial cells (77). Finally, Sulfs may also play an important role in cartilage formation. In quail, the highest expression level of QSulf-1 was observed in condensing mesenchyme, during the early differentiation stage of chondrogenesis. Interestingly, overexpression of QSulf-1 in quail micromass cultures enhanced differentiation of prechondrocytes into chondrogenic lineage, supporting its role in mesenchymal condensation and early differentiation of cartilaginous elements (65). In rat embryos, *in situ* hybridization showed a strong expression of Sulf-1 in the floor plate, choroid plexus, and cartilage (45).

### Role of the Sulfs in tissue homeostasis and repair

Besides their role during development, Sulfs have been shown to play various functions in homeostasis and repair of various tissues and organs. Sulfs are involved in muscle regeneration, by promoting satellite cells myogenic differentiation through disruption of FGF2 (80). Through their ability to modulate GDNF, Sulfs play a critical role in the maintenance of the spermatogonial stem cells (63). Sulf-2 has been shown to promote liver regeneration after partial hepatectomy, by modulating WNT3a and GLI1 signaling (81). Sulfs take part in cartilage homeostasis, by acting simultaneously as a positive and negative regulator of BMP and FGF activity, respectively (70). Interestingly, both Sulf-1 and -2 also showed enhanced expression in osteoarthritic and aging cartilage (82). In such tissues, resulting changes in HS sulfation pattern may alter its ability to modulate a variety of growth factors (FGF2, Wnt, BMP, Noggin), thereby contributing to abnormal chondrocyte activation and cartilage degradation (82). A study in quail also suggested a role in vascular homeostasis. Indeed, overexpression of QSulf-1 decreased adhesion, and increased proliferation and apoptosis of vascular smooth muscle cells, suggesting that optimal levels of HS 6-O-sulfation are critical to maintain proper functions of these cells (83). In addition, tuning of Sulf-1 expression has been proposed as a regulatory mechanism of leukocyte infiltration, by inhibiting binding of L-selectin and monocyte chemoattractant protein-1 (MCP-1) to vascular basement membrane HSPGs (84). Lastly, Kalus and colleagues provided evidence that the role of Sulfs in the nervous system extends beyond development, as these enzymes promote neuronal and behavioral plasticity in adults (61).

### Sulfs and diseases

Aside their multiple physiological functions, Sulfs have also been involved in a number of pathologies. Amongst these, the role of Sulfs in cancer has been by far the most studied and will be detailed below. In addition, overexpression of Sulf-2 has been observed in a murine model of Type 2 diabetes mellitus (85). Most interestingly, inhibition of the enzyme reduced post-prandial hypertriglyceridemia and restored VLDL-binding capacity of hepatocytes, thus highlighting Sulf-2 as an attractive therapeutic target to improve metabolic dyslipidemia (85). Recently, Sulf-2 overexpression has also been associated with idiopathic pulmonary fibrosis (IPF), most likely through the regulation of TGFβ1 signaling in Type 2 alveolar epithelial cells (86).

## Sulfs in Cancer: Intriguing and Ambivalent Functions

A wealth of evidence has reported that cell transformation and evolution through the different stages of malignancy is accompanied by modifications in HS expression and structure including changes in 6-O-sulfation (37, 38, 87). Therefore, when Sulfs emerged as a new way of post-synthetic editing HS 6-O-sulfation status, potential roles, and implication in cancer were extensively investigated.

Very early, the down-regulation of HSulf-1 was reported in ovarian cancer (72). Interestingly, re-expression of HSulf-1 in ovarian cancer cell lines reduced FGF2/HB-EGF signaling and cell proliferation *in vitro*, and increased sensitivity to pro-apoptotic drugs (72). Lowered levels of HSulf-1 expression were also observed in hepatocellular carcinoma (HCC), specific subtypes of breast cancer, clear cell sarcoma, kidney, gastric, and bladder cancers (72, 73, 88, 89), but other studies have since shown that HSulf-1 down-regulation is not ubiquitous in cancer. Original data from Lai et al. (72) have been disputed by Backen and colleagues, who reported a higher HSulf-1 expression in ovarian tumor cells than in normal cells (90), and overexpression of HSulf-1 has been broadly observed in cancers including leukemia, head and neck tumors, gastric and pancreatic cancers, brain tumors, invasive breast carcinoma and colon, pancreatic, esophageal, and lung adenocarcinoma (89, 91–94). In contrast, Sulf-2 has been consistently found over-expressed in tumors (89, 92, 94, 95).

Despite being closely related enzymes, misregulation of Sulf-1 and -2 have strikingly different consequences in cancer (89, 96, 97). Sulf-1 is largely reported as having a tumor suppressor activity, as described in HCC, myeloma, head and neck, breast, and pancreatic cancers (71, 73–75, 79, 98). Furthermore, it has been shown that expression of HSulf-1 in HCC and ovarian cancer cells enhanced the anti-tumor effects of pro-apoptotic drugs (72, 99, 100). On the contrary, Sulf-2 displays pro-oncogenic activity in HCC, pancreatic, breast, and lung cancers (94, 95, 97, 101, 102). Interestingly, silencing Sulf-2 expression in lung carcinoma cells resulted in loss of the transformed phenotype (102). In clinical surveys on multiple myeloma and HCC patients, Sulf-2 expression has been correlated with poor prognosis (91, 101). Finally, a role of Sulf-2 in the development of drug resistance was suggested on chronic lymphocytic leukemia cells (103). However, this general concept of two Sulfs with clear cut pro- and anti-oncogenic antagonist activities has been challenged in a number of recent studies. Overexpression of Sulf-2 has been shown to inhibit growth of myeloma tumors and breast cancer xenografts (71, 104), and Sulf-1 has been associated with poor prognosis in gastric cancer and lung adenocarcinoma (91, 92). Altogether, these conflicting data clearly highlight our poor understanding of the complex and multifactorial implication of the Sulfs in cancer.

One explanation may be that most studies on Sulfs were carried out on established cancer cell lines. Sulf action may thus differ in the context of clinical tumors, where distinct expression patterns of these enzymes within tumors could restrict their action to specific components of the tumor and its microenvironment. Furthermore, since Sulfs are extracellular enzymes, the paracrine activities of Sulfs expressed by other cell types of the tumor stroma and/or vasculature may also play a significant role. Regarding expression of the Sulfs, it is worth noting that in Quail, alternative splicing of QSulf-1 has been shown to generate a shorter isoform, QSulf-1B (105). Interestingly, full length QSulf-1 and QSulf-1B displayed antagonist activities, the latter inhibiting Wnt signaling and promoting angiogenesis. Although such splice variant has not yet been described for either human isoforms, the balance between their expression could further contribute to the diversity of Sulf function during cancer development.

Yet, much work is needed to clarify discrepancies between *in vitro* and *in vivo* observations and understand how two enzymes with highly similar catalytic activities can have opposite effects in tumor development. For example, Both HSulfs inhibits signaling of a number of growth factors *in vitro*, but display antagonist activities with regards to proliferation and angiogenesis *in vivo* (48, 55, 69, 73, 95, 98, 101). One obvious possibility is the existence of distinct substrate specificities. It is considered that processing of HS by the Sulfs results in a ~5–7% reduction in sulfate content. As discussed above, such discrete structural alterations have nonetheless great functional consequences. Therefore, even subtle variations in HSulf-1 or -2 specificities may be significant. Both HSulf-1 and -2 have been shown to act on HS [UA(2S)-GlcNS(6S)] and [UA-GlcNS(6S)] disaccharides, but the 6-O-desulfation of these disaccharide species is rarely exhaustive (48, 55, 56, 62). HS structural features could therefore be involved in selective recognition of the Sulfs, thereby influencing their action along the polysaccharide chain. As discussed above, there is no evidence yet suggesting differences in substrate recognition for the Sulfs. However, further investigation would be needed to clarify this particularly complex issue.

Another attractive hypothesis to explain such discrepancies is that Sulfs may exert their activity on spatially distinct substrates. Selective desulfation of cell surface HS would indeed result in a reduction of growth factor binding/activation and subsequent tyrosine kinase signaling, while desulfation of matrix HS may elicit release of growth factors from extracellular storage, thereby increasing their bioavailability for cell surface signaling receptors. In this context, the ability of the enzyme to diffuse throughout tissues might be a determinant factor: limited diffusion would restrict the enzyme to an autocrine activity at the cell surface, while rapid diffusion may allow access to more distant HS moieties, such as those having a storage function in the ECM. Sulf diffusion/bioavailability may be either controlled by the enzyme levels of expression, activity rates (efficient desulfation would speed up the bioavailability of the enzyme toward more distant substrates), HS-binding properties (residual binding to 6-*O*-desulfated HS might slow down the release of the enzyme), or binding to other cell surface/ECM components. A number of recent observations provide interesting but incomplete information regarding these hypotheses. Firstly, a study on HCC patients recently suggested a bimodal action of Sulf-1, related to its level of expression (97). Patients with tumors expressing intermediate levels of HSulf-1 showed better survival that those with low or high HSulf-1 expression. It could be speculated that a low amount of Sulf-1 is not sufficient for tumor suppressor activity through inhibition of pro-oncogenic and pro-angiogenic growth factor signaling, but that too high an expression causes additional alteration of ECM HS. Secondly, we have recently shown that, although HSulf-1 and -2 share a similar processive desulfation mechanism, they are characterized by distinct activity rates (55). Surprisingly, HSulf-1 displayed the highest activity. However, this analysis was carried out using a fully sulfated heparin oligosaccharide, and activity of HSulfs might be different in HS, where clusters of contiguous trisulfated disaccharides may be a rare occurrence. Thirdly, Frese and colleagues reported that HSulf-1 binding to 6-*O*-desulfated HS was dramatically decreased (48), thereby providing the basis for the release of the enzyme from its processed substrate. Further investigation will be needed to determine whether residual binding could still influence HSulf-1 diffusion, and whether this applies similarly for HSulf-2. Lastly, Milz et al. suggested that HSulf-1 could bind to CS/DS (68). As these GAGs are not suitable substrates for the enzyme, this interaction may limit diffusion of HSulf-1 throughout tissues.

Finally, it has been reported that Sulf activity influenced expression of other GAG related genes, thereby modifying GAG expression and structure. HSulf-2 has been shown to induce Glypican-3 expression (101). Moreover, differential expression of HS 2-*O*- and 6OSTs was reported in Sulf-1/2 knock-out mice, which correlated with variations in HS composition for Sulf non-substrate *N*-, 2-*O*-, and 6-*O*-sulfate groups (62). This observation suggests that Sulfs can modulate HS structure either directly, through their catalytic activity, or indirectly, by taking part in a feed-back regulatory mechanism of the polysaccharide biosynthesis.

## Sulfs as Targets for Anti-Cancer Therapeutic Approaches

HSulfs, and particularly HSulf-2, undoubtedly appear as attractive targets in cancer therapy. Sulf-2 overexpression and pro-oncogenic activity have been demonstrated for a number of tumors, including cancer of poor prognosis such as lung squamous cell carcinoma and lung adenocarcinoma (102). Sulf-2 therefore represents an interesting candidate as diagnostic and prognostic biomarkers, which are greatly needed, in particular for these cancers. Detection of HSulf-2 as a prognosis marker has already been achieved by monitoring gene expression or by immunochemistry (91, 93, 101). However, being extracellular enzymes, an interesting perspective would be the development of diagnostic/prognostic kits based on enzymatic assays performed on body fluids of patients, where the enzyme may accumulate.

Another advantage of HSulf-2 extracellular localization is that it can easily be targeted by low molecular weight compounds. Very recently, molecules in clinical development have shown Sulf-inhibiting activities. A disulfonyl nitrone derivative termed OKN-007 has been found to inhibit HSulf-2 and tumor growth in HCC (106). Interestingly, OKN-007 displays anti-glioma properties in a clinically relevant rat model (107) and safety data are already available for this molecule, which had previously undergone clinical trials as a treatment for ischemic stroke (106). In addition, proteasomal inhibitors have been found to suppress HSulf-2 expression in a number of cancer cell lines (96). These include FDA/EMEA approved drug Velcade (bortezomib), which was also shown to reduce tumor size, in MCF-10 derived mouse xenografts (96). However, mechanisms by which these compounds inhibit HSulf-2 are unknown and further investigation will be needed to establish the specificity of this activity.

A more rational approach to increase drug specificity is to develop HS mimetic inhibitors. The chemically sulfated polysaccharide PI-88 originally developed as an anti-heparanase agent has been found to also inhibit Sulfs (108). With regards to these activities, PI-88 has been tested in clinical trials for the treatment of lung and prostate cancer, but displays severe side effects (including immune-induced thrombocytopenia) because of its broad range of activities (109). Sulfamate-based compounds, notably aryl sulfamates, have been previously shown to inhibit a broad spectrum of bacterial and eukaryotic sulfatases (46). With this regard, Schelwies and colleagues recently designed sulfatase inhibitors corresponding to glucosamine-6-sulfamate analogs, which showed selectivity toward HSulfs (110). An interesting perspective pointed out by the authors would be to include such glucosamine mimetics within specific HS saccharide epitopes, in order to selectively target some signaling pathways. Finally, the development of monoclonal antibodies raised against HSulf-2 could lead to antibody-based therapies (93). Altogether, these data clearly emphasize the need of increasing specificity of drug candidates toward Sulf. Furthermore, with regards of the opposing activities of Sulf-1 and -2 in the development of many cancers, it could be of high interest to design inhibitors discriminating these two isoforms. However as discussed above, this would require further insights into the mechanism of action of these enzymes. Obtaining structural data on the Sulfs is another scientific achievement that would undoubtedly deliver crucial information for the development of biologically active inhibitory compounds. Structures of human sulfatases ArsA, ArsB, and ArsC have been solved by X-ray crystallography (111–113). However, solving the structure of the Sulfs, and in particular of their HD domains, may prove extremely complex, as these domains display no homology with any known proteins, are reported as poorly structured from *in silico* amino acid sequence analysis, and are particularly rich in (most probably) surface exposed charged amino acids, suggesting high protein flexibility.

## Conclusion

Tumor progression and metastasis are underpinned by mechanisms involving cell growth, dissociation, and migration, basement membrane degradation, invasion into the adjacent ECM, adhesion to the vasculature and extravasation/proliferation into metastatic sites. These are both cell- and tissue-driven processes that requires a vast arrays of information and molecular systems including complex signaling cytokine networks, adhesines, and stromal tissue remodeling proteases (114). Cell and matrix HS, which play a key role in orchestrating these signals, contribute at all stages in the tumor growth and metastatic process (115, 116). Most, if not all, of these molecular effectors (cytokines and chemokines that support tumor cells progression and migration, growth factors that promote angiogenesis, matrix metalo proteases involved in tissue degradation and remodeling, or adhesion molecules such as selectin) are HS-binding proteins. HSPG expression is developmentally regulated and altered in several pathological situations, including cancer. In that context, the Sulf enzymes that recently appeared to be importantly involved in the HS remodeling that characterize both tumor cells and surrounding stroma, add an additional level of regulation and complexity, and are likely to play key regulatory functions that remain to be elucidated.

## Conflict of Interest Statement

The authors declare that the research was conducted in the absence of any commercial or financial relationships that could be construed as a potential conflict of interest.
